# Real-Time Gauging of the Gelling Maturity of Duck Eggs Pickled in Strong Alkaline Solutions

**DOI:** 10.3390/foods10092057

**Published:** 2021-08-31

**Authors:** Ching-Wei Cheng, Kun-Ming Lai, Wan-Yu Liu, Cheng-Han Li, Yu-Hsun Chen, Chien-Chung Jeng

**Affiliations:** 1 College of Intelligence, National Taichung University of Science and Technology, Taichung 404, Taiwan; cwcheng@nutc.edu.tw; 2 Department of Health Industry Technology Management, Chung Shan Medical University, Taichung 402, Taiwan; quinn@csmu.edu.tw; 3 Department of Nutrition, Chung Shan Medical University Hospital, Taichung 402, Taiwan; 4 Department of Forestry, National Chung Hsing University, Taichung 402, Taiwan; wyliu@nchu.edu.tw; 5 Department of Bio-Industrial Mechatronics Engineering, National Chung Hsing University, Taichung 402, Taiwan; cghan@smail.nchu.edu.tw; 6 Department of Physics, National Chung Hsing University, Taichung 402, Taiwan; nessguang@gmail.com

**Keywords:** pickled eggs, transmittance, spectrometry, pickling

## Abstract

Although many ultraviolet-visible-near-infrared transmission spectroscopy techniques have been applied to chicken egg studies, such techniques are not suitable for duck eggs because duck eggshells are much thicker than chicken eggshells. In this study, a high-transmission spectrometer using an equilateral prism as a dispersive element and a flash lamp as a light source was constructed to nondestructively detect the transmission spectrum of duck eggs and monitor the pickling of eggs. The evolution of egg transmittance was highly correlated with the albumen during pickling. The transmittance exponentially decays with time during this period, and the decay rate is related to the pickling rate. The colors of the albumen and yolk remain almost unchanged in the first stage. A multiple linear regression analysis model that realizes a one-to-one association between the days of pickling and the transmission spectra was constructed to determine the pickling duration in the second stage. The coefficient of determination reached 0.88 for a single variable, wavelength, at 590 nm. This method can monitor the maturity of pickled eggs in real time and does not require the evolution of light transmittance.

## 1. Introduction

Eggs are nutritious and rich in protein and amino acids [1]. When eggs are stored at room temperature, their freshness gradually decreases over time. In Asia, duck eggs are marinated to prepare pickled or salted eggs using strong alkaline liquids. In addition to increasing the flavor of eggs, this process considerably extends their shelf life. Particularly, pickled eggs can be stored at room temperature for more than 6 months. Pickled eggs are called “century eggs” or “thousand-year eggs” by westerners, reflecting their long-term storage. Pickled eggs have a distinctive pungent aroma that may cause nausea for some consumers; however, they are very popular in Asia. According to statistics from the Taiwan government, Taiwan’s annual output of duck eggs is ~500 million units. The majority of duck eggs are processed into pickled or salted eggs. These processed eggs are very popular and are used in traditional delicacies such as pickled egg salads, pickled egg tofu, and fried pickled eggs. Pickled eggs may accumulate iron (Fe), zinc (Zn), and other minerals during the marinating process, and most of their proteins and fats are broken into various peptides, free amino acids, and free fatty acids by strong alkalis. Thus, they have less fat [2]. Pickled egg is a traditional Chinese meal. Additionally, it is considered among Chinese medicines and can be used as a functional food for anti-inflammatory therapy [3].

Duck or quail eggs are mostly selected for preservation because of their thick shells. The eggs are immersed in strong alkaline liquids or alkaline mud to mature. Such strong alkalis slowly permeate through the eggshells and eggshell membranes of the eggs, causing the proteins of the egg whites to undergo a series of chemical changes and gradually changing the albumen’s gel-like structure [4]. The immersion method entails marinating pickled eggs with salt, tea, and other substances that are usually mixed with sodium hydroxide or potassium hydroxide in specific ratios [5]. To adjust the osmotic pressure of the alkaline solution used in pickling eggs and increase the success rate of the preserving process, minute amounts of metal salt compounds are added to the pickling solution [4,5,6]. Using this method, the time required to produce pickled eggs depends on the concentration of the alkaline solution, the type and concentration of the used metal compounds, and the temperature of the pickling environment. The marinating process is tedious and can take weeks or months. However, it usually requires up to 3 months. If the concentration of the alkali liquor is considerably high or the eggshell is slightly cracked, the strong alkalis can enter the eggs, causing the gelatinous albumen to be hydrolyzed and liquefied and resulting in pickling failure [7,8]. Eggshell quality is extremely important in pickling eggs. Traditionally, percussion methods are used for crack detection [9]. Fully intact and thick eggshells result in pickled eggs with better quality compared with weak eggshells. Moreover, thick and intact eggshells make it more likely for white amino acid crystals, which are known as pine-floral crystals in China, to precipitate in the albumen, which is necessary for the preserving process. Appropriate concentrations of metal oxides are added to the alkaline marinade to form insoluble sulfur compounds, which can plug the shell and membrane pores and mesh the corrosion holes produced during marination [8,10,11]. The addition of metal ions prevents the excessive infiltration of alkalis, thus improving the success rate and quality of egg preservation. Traditionally, adding lead oxide worked best; however, the health hazards related to the lead content in marinated eggs have been a problem in the past. Lead is strictly prohibited in Taiwan, and residual lead in eggs cannot exceed 0.3 ppm. There are already alternatives to lead oxides [8], such as copper sulfate (CuSO_4_), zinc sulfate (ZnSO_4_), ferric sulfate (Fe_2_(SO_4_)_3_), magnesium chloride (MgCl_2_), and calcium chloride (CaCl_2_) [8]. However, these compounds reduce the stability and quality of pickling. Nondestructive monitoring of the production process is critical for improving the marinating process and is important for reducing the incentives of using lead oxide.

Visible light or near-infrared spectroscopy is often used in the nondestructive monitoring of the protein quality and freshness of egg products [12,13]. The light absorption ratios of different egg components, such as erythrocytes, shells, egg whites, and blood vessels, can determine whether an egg is fertilized [14,15]. Erythrocytes are developed during the growth of embryos in eggs and exhibit different absorption spectral bands [16]. Using the optical detection method, which is similar to the machine vision, one side of the egg is illuminated by a strong light source. Then, a charge coupled device (CCD) camera is placed on the other side of the egg to determine the spectral absorption wavelengths, and thereby the egg’s fertilization and development status. Using a 50 W tungsten halogen lamp to illuminate eggshells and a near-infrared (NIR) hyperspectral imaging system, Liu and Ngadi (2013) collected spectral images in the 900–1700 nm wavelength range. A Gabor filter was applied to the region of interest in each hyperspectral image and the texture information was extracted from this region. This information was then used to determine the fertility and early embryo development of chicken eggs [17]. Generally, pickled eggs are tapped with a finger to detect the amount of oscillation and determine their gel state, which is not an objective method. Moreover, an accelerometer can be used to detect the oscillation decay rate and determine gel quality [18]. However, presently, there are no appropriate scientific methods for monitoring the changes occurring in pickled egg gels during pickling.

In this study, aiming to replace the dispersion element grating with equilateral prisms, improve the efficiency of light utilization, and develop a large-aperture optical system, we used a Xe flashbulb. Then, for the first time, the transmission spectrum of duck eggs was successfully obtained, and the duck egg pickling process was observed in a nondestructive manner. Furthermore, the proposed system provided quantitative indicators, such as penetration intensity variations and color changes, and the spectral transmittance of the eggs significantly increased in the first week before exponentially decaying over time. The transmittance rising rate, transmittance compared with that of the zeroth day (T/T_0_), and attenuation rate (half-life t_1/2_) could be used as objective indicators of pickling efficiency. The penetration spectrum was normalized using the standard normal variate (SNV) method, through which the color shade was removed, thus leaving only the color change message. A multiple linear regression analysis (MLR) of the spectral transmittance was performed in a single wavelength, and it was sufficient for calculating the number of days of marination. Furthermore, the discoloration rate could be used to quantify the degree of marination.

## 2. Materials and Methods

### 2.1. Egg Samples and Marinade

In this study, 115 unfertilized *Anas platyrhynchos* (duck) eggs were used to prepare pickled eggs. The formula used for the pickling solution was 100% water, 3.5% sodium hydroxide, 10% table salt, 0.1% zinc sulfate, and 0.1% ferrous sulfate. The pickling average room temperature was 25 °C.

Forty-five of the duck eggs were used to make pickled eggs, and the changes that occurred in the egg gels during the pickling process were monitored. The eggshells were removed, images were taken, and the changes inside the eggs were recorded. The other 70 duck eggs were recorded for spectral transmittance measurement. The transmittance rate rapidly increased in the first seven days of the pickling and exponentially decayed after seven days. Therefore, the subsequent rate of change was slow, and the testing time was gradually extended once every two days, twice a week, and once in the last week. The detection days were day 1 to 7, 9, 12, 16, 20, 24, 29, 35, and 42 after the start of pickling. In another experiment, we prepared 30 fresh duck eggs and purchased 60 preserved eggs produced by two different manufacturers and verified them by the transmittance spectrum established in this article.

### 2.2. Preliminary Experiment

Many ultraviolet (UV)-visible (Vis)-NIR transmission spectroscopy techniques have been employed in chicken egg studies to inspect blood spots, freshness [19,20,21], and egg yolk contents [22]. However, UV-Vis-NIR transmission spectroscopy is not suitable for duck egg inspection because duck eggshells are much thicker than chicken eggshells, causing the transmission spectra to be too weak and noisy. Tungsten halogen lamps without characteristic spectral lines are commonly used as light sources for spectroscopic detection. Because duck eggshells are usually colored, they are more efficient in turning absorbed light into heat compared with chicken eggs. Nevertheless, to enhance transmission signals by increasing the power of halogen light sources, the eggs are heated using bright light, resulting in protein structure damages. Experiments were conducted with halogen lamps as light sources. No correct penetration spectra can be measured before the duck eggs are cooked by high-intensity light. In particular, with a halogen lamp as a 3000 K black body, the energy of the band from 500 to 800 nm, which is used in the spectral measurements, is only 13% of the total spectrum. If absorbed, the rest of the band is transformed into thermal energy, causing the egg to warm up and get damaged. Therefore, to resolve the dilemma caused by the heat damage of intense light, a series of major improvements are still needed.

### 2.3. Experimental Setup and Calibration

**Figure 1:**
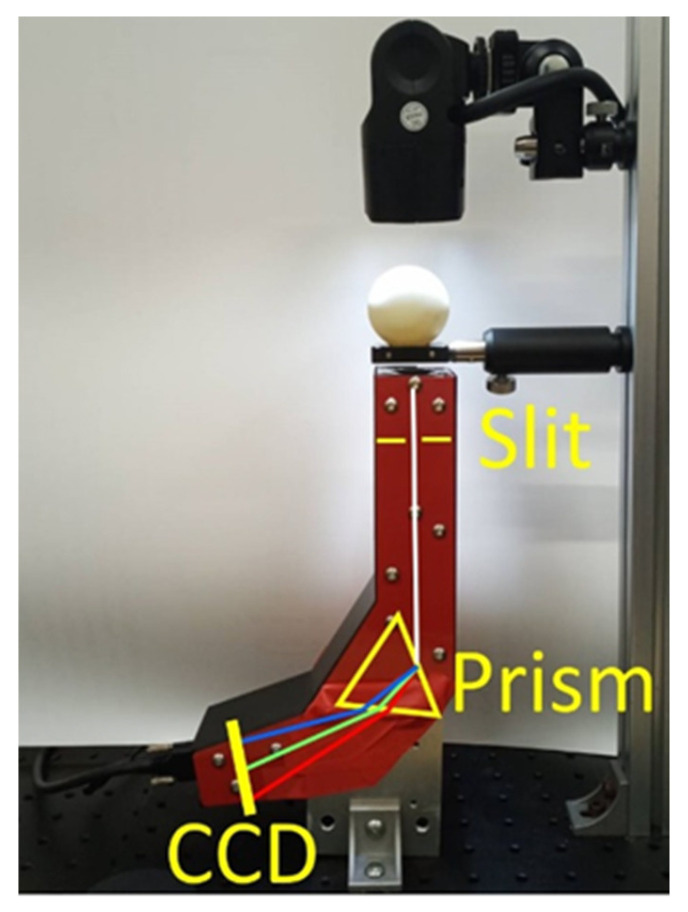
Duck egg transmission measurement spectrometer. The xenon flash is located at the top as the light source. Light passes through the lens and then enters the inhouse optical prism spectrometer at the bottom after penetrating the egg. The “CCD” in the figure means “charge coupled device”.

**Figure 2:**
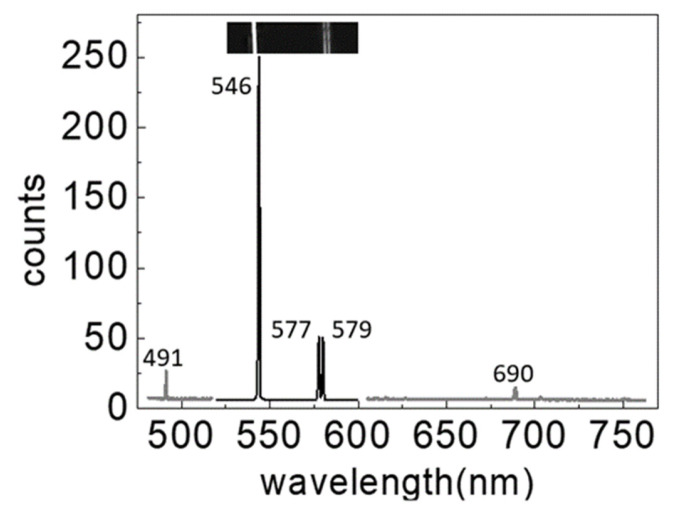
The inset shows a photo of the mercury lamp spectral lines, in which three bright lines appear at 546, 577, and 579 nm. The characteristic spectral lines of the mercury lamp in terms of wavelength are converted by interpolation. The darker portion in the middle is obtained with an exposure time of 1/125 s, and the gray portions on the two sides are obtained with an exposure time of 1/15 s. The spectral lines at 577 and 579 nm are distinguishable, showing that the resolution is less than 2 nm.

Figure 1 shows the settings of the used transmission measurement equipment. The light from the Xe flash is focused using the lens, passes through the egg, and then enters the inhouse light prism spectrometer below. Then, the transmission spectrum of the duck egg is measured. An inhouse spectrometer comprising equilateral dispersive prisms as spectroscopic devices, having a slit width of 10 µm and a CCD imaging sensor (ICX274, Sony, size type: 1/1.8, 1600 × 1200 pixels, Tokyo, Japan) was used as a light detector. The spectrometer was calibrated using a Hg lamp (Oriel Instruments, Mercury Lamp 6034, Stratford, CT, USA). The locations of the Hg–Xe characteristic spectra on the CCD imaging sensor were then recorded (see the inset at the top of Figure 2). Afterward, through interpolation, the pixel locations of the incoming light on the CCD sensor were converted into the corresponding wavelengths (Figure 2). In the figure, the characteristic wavelengths are 491, 546, and 690 nm, and the lines at 577 and 579 nm can be clearly distinguishable. The effective resolution is less than 2 nm. The spectrometer performed measurements from 480 to 820 nm (1600 pixels), with each pixel corresponding to ~0.1–0.35 nm. Although the specular separation capability of the prism is less than that of the gratings and does not provide a high resolution, the green light area (~100 nm) is only deflected by 2°, where the transmittance rate of the prism can reach 90%. Although the resolution of the reflective diffraction grating is better than a prism, the average reflectivity is only ~50%. The duck egg transmittance rate is very low, and the spectrum is dull. Further, the prism can be used to reduce the exposure time or light source intensity.

### 2.4. Transmittance Measurement

When halogen lamps are used as light sources, heat damages to egg proteins are reported in almost all eggs, resulting in incorrect spectral transmittance. After measurement, duck eggs are cut open, and their proteins are reported to be clear and transparent with no heat deteriorations. In this study, a common photographic Xe flash was used as a light source for measuring the transmission spectrum of pickled duck eggs.

The Xe flash lamp was flashed three times. After its light passed through each duck egg sample, the wavelength spectrum was detected using the CCD sensor. Afterward, to confirm that the eggs had not been damaged by the heat of the light source, the shells of the 45 duck eggs that were used in the experiment were removed to establish a spectral model. Meanwhile, previous experiments have demonstrated that duck eggs are not damaged by light sources of this type. A high-intensity flash ensured the good penetration of the used light; however, the pulse was extremely short. Therefore, in this setup, Xe lamps do not generate sufficient heat to destroy egg proteins.

**Figure 3:**
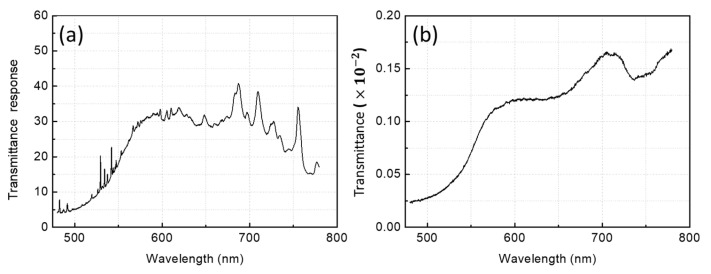
(**a**) Transmission spectrum of a duck egg using xenon flash as a light source. (**b**) Transmittance of a duck egg obtained by dividing the egg transmission response by the spectrum of the light source.

Figure 3a shows the transmission spectrum obtained by the 200 W xenon flash. The spectrum has a convex shape mixed with sharp peaks, where the convex shape is attributed to the optical system, while the peaks are obtained from the xenon flash. The transmittance of the duck egg, as shown in Figure 3b, was obtained by dividing the egg transmission response by the light source’s spectrum. Consequently, the characteristic spectral line of the Xe source, as well as the uneven efficiency of the optical system, was completely removed.

### 2.5. Statistical Analysis

The spectrum was normalized using a SNV (Equation (1)). The resulting spectrum was then obtained by subtracting the average value and then dividing it by the standard deviation. The SNV process then removes the light shade of the spectrum and shows only the color characteristics [23,24].

(1) $$nT_{i\lambda }=\frac{T_{i\lambda }-{\bar{T}}_{i}}{SD_{i}}$$

where $nT_{i\lambda }$ is the normalized transmittance of the wavelength λ on the *i*th day, $T_{i\lambda }$ is the original transmittance of the wavelength λ on the *i*th day, ${\bar{T}}_{i}$ is the average transmittance spectrum on day I, and $SD_{i}$ is the standard deviation of the transmittance on the *i*th day.

Then, to evaluate the degree and effect of pickling, the normalized spectrum was analyzed, and a regression model was established to predict the corresponding variables using MLR to determine the relationship between the pickling days and the spectral band of the transmittance. Regression analysis is a type of statistical analysis that is used to determine the effect of independent variables on dependent variables [25]. Additionally, it is used to evaluate the collinearity between variables and in making predictions. This technique has been extensively applied in agriculture, and it was used in this study to identify the relationship between the protein gel changes in the pickled eggs, transmission spectra, and number of pickling days [19].

Regression analysis evaluates the y results based on the x value and error value. The established prediction model based on the performed linear regression is demonstrated by Equation (2), where $D_{p}$ is the predicted pickling days, and $nT\left(\lambda_{i}\right)$ is the ith unknown regression coefficient. Using Equation (2), an analysis of the variance was performed to obtain the determination coefficient, R^2^ (0 < R^2^ < 1), with a significance comparable with the percentage of the total explainable variance in the regression model. The closer R^2^ is to 1, the stronger is the collinearity.

(2) $$D_{p}=\beta_{0}+\beta_{1}\times nT\left(\lambda_{1}\right)+\beta_{2}\times nT\left(\lambda_{2}\right)+\ldots +\beta_{i}\times nT\left(\lambda_{i}\right).$$

## 3. Results and Discussion

### 3.1. Observation of the Eggshell Removal

**Figure 4:**
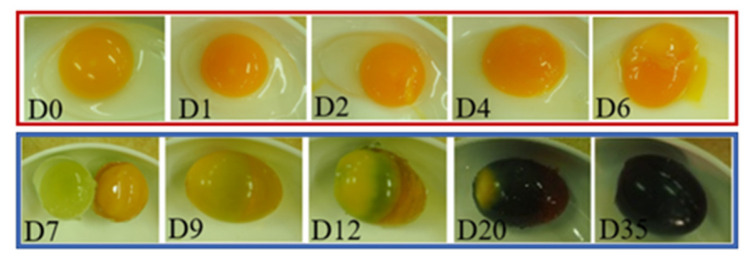
Evolution of albumen and yolk during pickling. They gradually solidified in the first six days and then began to discolor after a week. The “D” in the figure means “day”.

During the 6 weeks of marination, the shells of a few eggs were removed for observation. The albumen gradually turned into gelatin under the action of a strong alkali. Then, the color started to turn yellow and finally turned dark brown. The entire process can be roughly divided into two stages:

- Albumen gelation stage: In the first week, the albumen and yolk gradually gelatinized, but their colors did not change. The albumen remained clear and transparent, and the boundary between the thick and thin egg white gradually disappeared. After about a week, the albumen was completely gelatinized. The outer layer of the yolk solidified, while the inner layer remained mostly liquid (Figure 4).
- Darkening and discoloration stage: After a week, the color of the protein gradually darkened and finally became dark brown, as shown in Figure 4. This darkening was a result of the reaction between the amino acids of the decomposed proteins and the reducing sugars [20] in the egg. The reaction was slow at room temperature and took several weeks. Generally, the process is faster in summer because of the high summer temperatures.

The conditions for producing pickled eggs are stricter than those for producing salted eggs. It is necessary to maintain complete eggshells throughout the production process, and the pickling conditions must be controlled to prevent incomplete gelation or over-marination, which reliquefies egg whites. Incorrectly marinated pickled eggs have a strong pungent smell and are not suitable for human consumption.

The finger-tapping detection method is currently the main method used in factories for inspecting pickled eggs. Traditionally, the manual inspection of pickled eggs is performed by tapping a finger on the eggshells. A pickled egg with good gelation usually oscillates after finger tapping, but those that are liquid or incompletely gelled will not oscillate [5,18]. All the 70 duck eggs used in the experiment passed the tapping examination, indicating that they were all successfully pickled.

In this study, the eggs were opened to observe whether they were gelled, and all the eggs were completely gelled.

### 3.2. Modeling of the Transmittance and the Marination Processes

**Figure 5:**
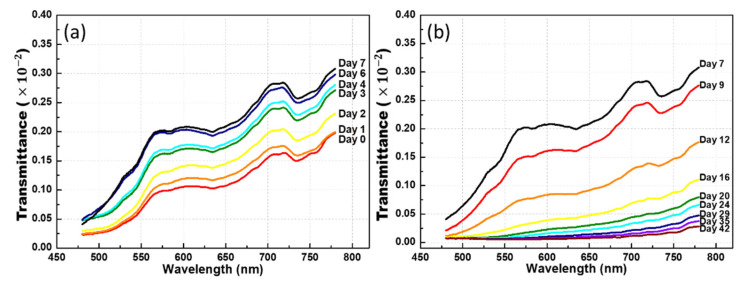
Average transmittance over the 70 duck eggs during the pickling process. (**a**) The average transmittance approximately doubled in the first week, and the standard deviation (SD) was 0.34. (**b**) From days 7 to 42, the transmittance gradually decreased.

During the marination process, the color of the albumen gradually changed from transparent to dark brown. However, as expected, the transmittance did not decrease with time. As shown in Figure 5a, the average transmittance significantly increased in the first week of solidification. In the 590 nm band, the average transmittance was approximately doubled, and the standard deviation (SD) was 0.34. These changes may be related to the albumen gradually becoming transparent and eggshell gradually becoming thinner because of the interaction with the strong alkali during the curing process. As shown in Figure 5b, the transmittance gradually decreased after one week. The decrease in transmittance was consistent with the gradual darkening of the egg white and egg yolk.

**Figure 6:**
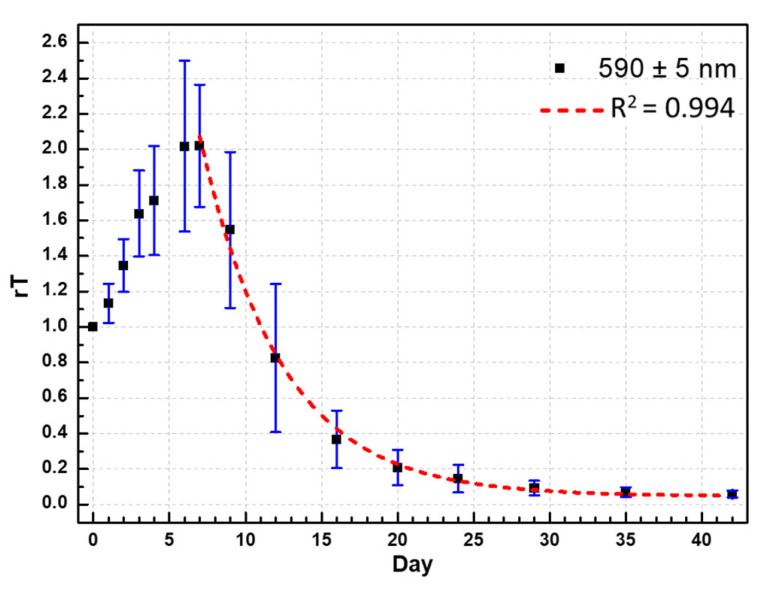
Relative transmittance (relative to the zeroth day), rT, of the duck eggs in the 590 ± 5 nm band. rT considerably increased by approximately two times at the end of the first week and then decreased. The red line is the exponential decay model curve.

Figure 6 shows the duck egg transmittance compared with that of the zeroth day (rT = T/T_0_) in the 590 ± 5 nm band. rT significantly increased in the first week and was at the maximum after 6.3 days, with an SD of 1.6 days. The maximum relative transmittance, rT_Max_, had an average value of 2.02 with an SD of 0.34. The relative transmittance decreased after a week. An exponential decay model (Equation (3)) was developed and used to predict the relative transmittance on the seventh day. The experimental measurement fits the model curve of Equation (3) with the coefficient of determination R^2^ = 0.885, as shown in Figure 6. In the marination experiment, the half-life, t_1/2_, of the decrease in transmittance was 3.72 days, the SD was 1.08 days, and the coefficient of determination was 0.994. In other words, the color became twice as dark every half a week. This darkening may be attributed to the amount of glucose present in the egg albumen. Maillard’s reaction links the carbonyl group of the reducing carbohydrates, amino group of the free amino acids, and lysine residues in the proteins (decomposed by the strong alkali) [26,27,28,29]. Furthermore, the darkening may have been caused by the reaction of the amino acids with Fe or S. Both cases are related to alkaline concentration and temperature. Thus, the half-life, t_1/2_, of pickled eggs should differ between winter and summer. t_1/2_ is a quantitative index and can be used to adjust the pickling time and marinade ingredients to avoid excessive pickling and pickling failures.

(3) $$\mathrm{rT}=rT_{\mathrm{Max}}\cdot 2^{-\frac{\left(Day-7\right)}{t_{1/2}}}+0.048$$

where rT: T/T_0_, relative transmittance (relative to that on day zero); rT_Max_: maximum relative transmittance of rT; t_1/2_: half-life of rT.

Through this study, it was found that the larger the value of rT_Max_, the longer is the half-life t_1/2_. These two quantities are significantly positively correlated (Pearson’s *r* correlation coefficient is 0.303) with a significance of 0.01. The mechanism of this correlation needs to be explored further. Moreover, it was found that the greater the first-day penetration rate T_0_, the shorter the half-life t_1/2_. These two quantities are significantly negatively correlated (Pearson’s *r* correlation coefficient is −0.27) with a significance of 0.023. A larger T_0_ may be attributed to a thinner duck eggshell and a smaller egg. The ingredients in the marinade easily penetrated the eggshells and diffused into the eggs. The pickling reaction is thus faster, and darkening takes less time.

### 3.3. Standard Normal Variation and Multiple Linear Regression of Transmittance

**Figure 7:**
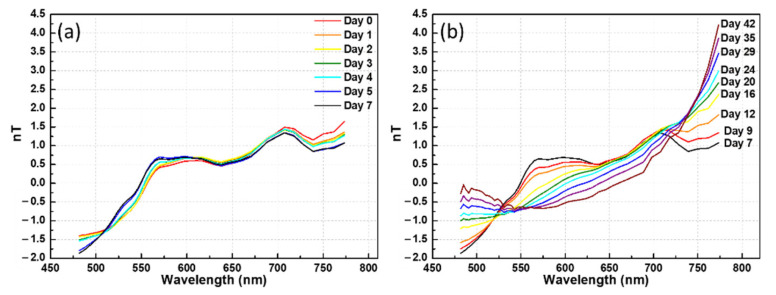
Normalized transmittance, nT, obtained by the standard normal variate: (**a**) 0–7 days and (**b**) 7–42 days.

The normalized transmittance spectrum (nT) was obtained by SNV (Equation (1)), which removed the color shade difference, thus only leaving the color message. The 1500 data points in the spectral range from 480 to 780 nm were reduced to 50 bands by averaging every 30 data points, where the range of each band was 4–10 nm. In the first week, the nT values of each day almost coincided, as shown in Figure 7a. The absorption characteristics of the spectrum did not change, i.e., the color did not change. These results are consistent with the observation after the eggshell was removed, i.e., the albumen remained transparent, and the yolk color remained unchanged in the first week. Later, as shown in Figure 7b, not only did the transmittance decrease but the spectral absorption characteristics also changed. This again is consistent with the observation made after the eggshells were removed, i.e., the color of the albumen gradually changed from transparent to brown, and the yolk turned from yellow to dark green.

In this study, the measured transmittance spectra of 70 duck eggs in the pickling process were normalized. Then, the relationship between the normalized transmittance spectrum (*λ_n_*) and marinating time after one week was analyzed by MLR, and a calibration curve was established [30,31]. Table 1 lists the correlation coefficient R, determination coefficient R^2^, and characteristic wavelength λn of the three modes. The three characteristic wavelengths are 590, 740, and 655 nm. Equations (4)–(6) show the relationship between the predicted days, $D_{p}$, and nT(*λ_n_*) values of the three modes:

(4) $$D_{p}=-23.3\times nT\left(\lambda_{1}\right)+21.3;$$

(5) $$D_{p}=-29.5\times nT\left(\lambda_{1}\right)-8.63\times nT\left(\lambda_{2}\right)+34.6;$$

(6) $$D_{p}=-38.1\times nT\left(\lambda_{1}\right)-13.6\times nT\left(\lambda_{2}\right)+8.5\times nT\left(\lambda_{3}\right)+39.1.$$

The coefficient of determination R^2^ reached 0.88 even with a single variable (590 nm band).

Because the pickling days in the record are labeled by integers D and the dates $D_{p}$ predicted by the three modes are generally nonintegers, the two should be linearly related using $D_{p}=aD+c$. In the ideal case, the slope a should be 1 and the constant c should be 0. Hence, in the three modes, $D_{p}$ and D from a single variable to three variables are related to

(7) $$D_{p}=0.88\times D+2.67,$$

(8) $$D_{p}=0.90\times D+2.12,$$

(9) $$D_{p}=0.92\times D+1.71.$$

The slope of the line increased from 0.88 and 0.90 to 0.92, corresponding to the errors of 12%, 10%, and 8%, where the constant terms are 2.67, 2.12, and 1.71. According to Table 1, for the spectrum analyzed by MLR, using a single wavelength of 590 nm, the coefficient of determination became 0.88. Upon using two wavelengths of 590 and 739 nm and three wavelengths of 590, 740, and 655 nm, the coefficient of determination as well as the prediction accuracy of the number of days of marination increased. In the case of the 70 pickled duck eggs, the coefficient of determination using a single wavelength of 590 nm was 0.88, which has a certain detection effect. Therefore, by only using a single wavelength, the number of days for which a duck egg is marinated can be successfully predicted.

**Figure 8:**
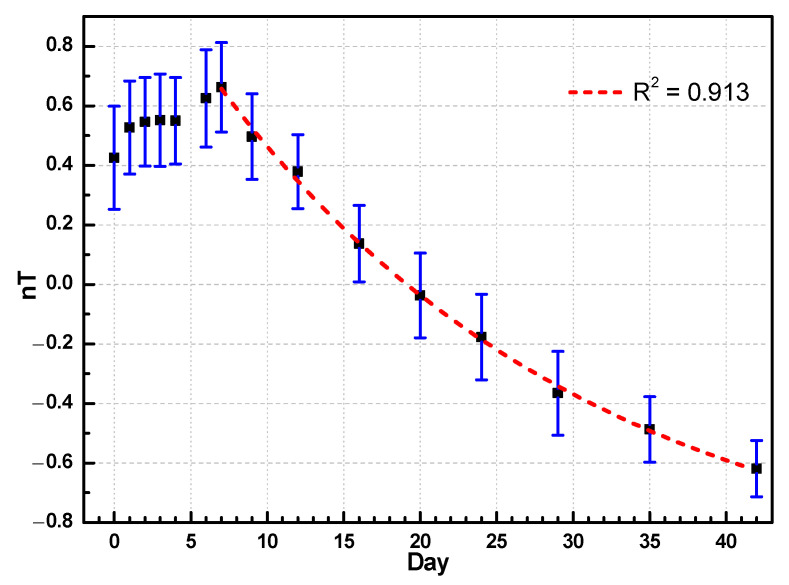
Evolution of the normalized transmittance spectrum, nT(*λ*_1_), in the 590 nm band. nT(*λ*_1_) changed slightly in the first week and then exponentially decayed. The value then dropped from 0.66 to −0.62 over five weeks.

Figure 8 shows the evolution of the normalized transmittance spectrum nT(*λ*_1_) obtained using the SNV method in the 590 nm band. The nT(*λ*_1_) value changed slightly in the first week, which is consistent with the direct visual observations of the albumen and yolk after the shell was removed. The situation after a week was nonlinear but properly fit the exponential curve. Compared with the exponential decay model in Equation (10), the R^2^ value of the 70 duck eggs was 0.913, and the half-life of the normalized transmittance was 16.6 days. Following Equation (10), $D_{p}$ is related to the actual days D, as $D_{p}$ = 1.03 × D + 0.32. The slope of the relationship is 1.03 (with an error of 3%), and the constant is 0.32. Compared with the MLR curve, the slope was close to 1 and the constant term was smaller.

(10) $$nT\left(\lambda_{1}\right)=1.67\times 2^{-\frac{\left(\mathrm{Day}-7\right)}{16.61}}-1.01,$$

nT(*λ*_1_) did not significantly change from day 1 to 6 (the values all fell within 0.559 ± 0.038). After the first week, the value monotonically decreased from 0.66 to −0.62 over five weeks. The distinctive value distribution of nT(*λ*_1_) with time makes it a viable index for estimating the degree of pickling. For example, if it is above 0.5, this indicates that the eggs are in the early pickling stage. Moreover, a value near 0 indicates that the egg is in the middle pickling stage. If the value of nT(*λ*_1_) is below −0.4, the egg is close to the completion of pickling and the marinating process should be terminated to avoid excessive pickling. This method can then be easily applied without knowing the value on the first day.

**Figure 9:**
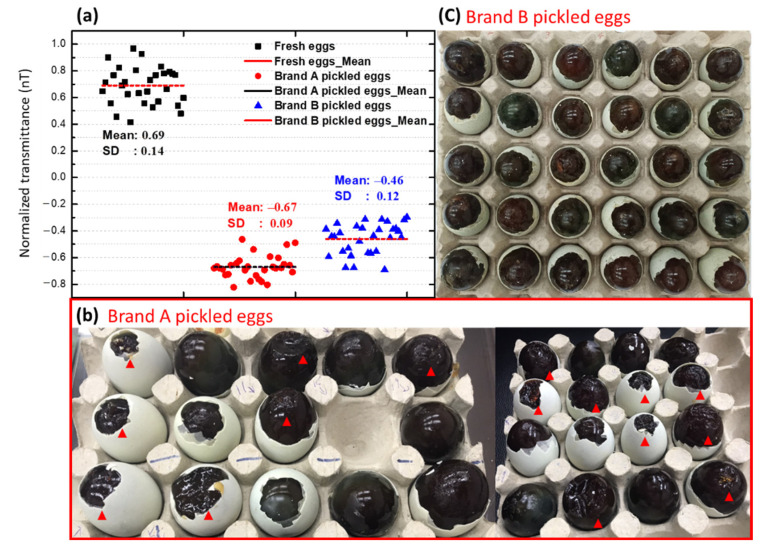
(**a**) 590 ± 3 nm band normalized transmittance spectrum validation scatter diagram of fresh duck eggs and commercially available preserved eggs. (**b**) Brand A preserved eggs after the removal of the shell (the red triangle is marked as gel failure). (**c**) Brand B preserved eggs after the removal of the shell, and all preserved eggs were successfully gelled.

To validate the degree of the pickling process of duck eggs, we obtained 30 fresh duck eggs and 30 pickled eggs from two different manufacturers (Factory A and Factory B) and measured the normalized transmittance (nT(*λ*_1_)) at 590 ± 3 nm. As shown in Figure 9a, the average (nT(*λ*_1_)) of pickled duck eggs from Factory A and Factory B was −0.67 (SD: 0.09) and −0.46 (SD: 0.12), respectively. Both of these values are lower than or close to that (−0.40 ± 0.09) recommended by us. The removal of the eggshells of 30 pickled eggs from Factory A revealed that the pointed end of 53% of the eggs was hydrated or dissolved (Figure 9b). The normalized transmittance (nT(*λ*_1_)) of most preserved eggs of Brand A is less than −0.6. In contrast, removal of the eggshell of 30 pickled duck eggs from Factory B revealed that the pointed end of the eggs was smooth and complete in shape. The normalized transmittance (nT(*λ*_1_)) of Brand B is mainly distributed at −0.4. It is unclear why the shape of the pointed end of the pickled eggs differs between the two factories, but the difference in pickling time (30–40 days) [5] may be responsible for the discrepancy. Therefore, future studies should focus on minimizing the hydration and dissolution of the pointed end of pickled duck eggs by determining the best pickling time for duck eggs. Nevertheless, these results indicate that the level of the red green blue (RGB) spectra parallels well with the experimental results, and thus it is possible to predict pickling levels of duck eggs using RGB spectra.

The limitation of this study is that Xe flash must be used as the light source for measuring the transmittance of duck eggs. In addition, in the measurement, a shading cloth is used to cover the measurement equipment to avoid interference from the surrounding environment light source that affects the measurement accuracy during the detection process. Suppose the transmittance is used to gauge the maturity of preserved eggs during the pickling process. In that case, the transmittance of fresh duck eggs is measured before pickling and compared with that of the pickled duck eggs. The transmittance rate on day 30 was only ~10% of that on day 1, which is close to the completion of pickling. The standard normal variation of transmittance nT(*λ*_1_) is used to judge the degree of maturity in real time based on the change in color, and the maturity can be judged without comparing the conditions on the first day. The longer the marinating time, the higher the degree of maturity, and the smaller was nT(*λ*_1_). When nT(*λ*_1_) reached −0.4 (~30 days), marination was complete. This method can be used to determine the maturity of white-shell eggs but is less suitable for the evaluation of brown-shell eggs or quail eggs. The color of the eggshell causes interference and affects the accuracy of the maturity determination.

The traditional method of judging maturity involves removing the eggshell and observing the color changes of the egg albumen. The darker the albumen color, the higher is the maturity. After pickled eggs are marinated for a week, as the marinating time increases, the transmittance decreases (Figure 5b). The lower the transmittance, the higher is the degree of maturation. Any wavelength can be chosen to observe the changes in transmittance, e.g., 590 nm. As shown in Figure 6, the transmittance rate at day 30 was only ~10% that of the first day, which is close to the completion of pickling.

In addition, it was found that the colors of the pickled egg albumen and egg yolk are related to the degree of maturation. After marinating for a week, the longer the marinating time, the higher is the degree of maturity. The albumen changed from colorless to glutinous to dark green, but the color change could be roughly distinguished by the naked eye. The SNV process then removed the light shade of the spectrum and only showed the color characteristics; therefore, the normalized transmittance nT had obvious changes, as shown in (Figure 7b). It can be seen from the graph that the normalized transmittance nT significantly varied with the number of days in 550–600 nm wavelengths. The nT(*λ*_1_) at 590 nm changed a little in the first week and then exponentially decayed. The value then dropped from 0.66 to −0.62 over six weeks. The longer the marinating time, the higher the degree of maturity, and the smaller the value. When nT(*λ*_1_) reached −0.4 (~30 days), marination was complete. The study can be used for real-time gauging of the gelling maturity of duck eggs pickled in alkaline solution and can be applied without comparing the first-day transmittance.

## 4. Conclusions

In this study, a novel high-transmittance spectrometer was developed and used to successfully observe the spectral transmittance of duck eggs and perform nondestructive monitoring of the pickling processes of pickled eggs for the first time. The evolution of the transmittance of eggs is correlated with the change in the albumen form during pickling. An MLR analysis was performed, and it yielded the relationship between the normalized spectral transmittance, nT(λn), and marinating time. In addition, a single-variable (590 nm band) linear regression was performed, and it yielded a result with a coefficient of determination, R^2^, of 0.88. From 1 to 6 days, nT(*λ*_1_) did not change much (~0.559 ± 0.038). One week later, the value monotonically decreased from 0.66 to −0.62. Overall, nT(*λ*_1_) was sufficient for estimating the pickling degree.

## Figures and Tables

**Table 1:** Correlation coefficient R, determining coefficient R^2^, and characteristic wavelength $\lambda_{n}$ of the three modes of the linear regression of the pickling days as a function of the standard normal variate (SNV) transmission rate.

Model	R	R^2^	*λ_n_* (nm)
**1**	0.94	0.88	590
**2**	0.95	0.90	590, 740
**3**	0.96	0.92	590, 740, 655
